# 

**Published:** 1888-08-25

**Authors:** 


### "THE HOSPITAL" LIBRARY OF NEW AND STANDARD WORKS.

For all information respecting the appearance of Titles, Prices, etc., of Books in this page, apply to Mr. W. H. HOWE, office of THE HOSPITAL, 38, Old Jewry.

### Medical Library.

ABDOMEN; Diseases of the. By S. O. Habershon. 21/- *Churchill.*
CANCER: Its Allies, and other Tumours. By F. A. Purcell. 10/6 *Churchill.*
DIPHTHERIA: Its Nature and Treatment. By Sir Morell Mackenzie. 5/- *Churchill.*
EAR: The Diseases of the. By A. Hartmann. 9/- *Pentland.*
EYE: On Diseases of the. By C. B. Taylor. *Redway.*
HEART: A Practical Treatise on Diseases of the. By W. H. Walshe. 16/- *Smith and Elder.*
HELPS to Health. By Hy. C. Burdett. 1/6 *Kegan Paul.*
HELP in Sickness. By Hy. C. Burdett. 1/6 *Kegan Paul.*
LIVER: Diseases of the. By Geo. Harley. 21/- *Churchill.*
LUNGS: A Practical Treatise on Diseases of the. By W H. Walshe. 16/- *Smith and Elder.*
MIDWIFERY FOR MIDWIVES. A Manual of. By Fancourt Barnes. 6/- *Smith and Elder.*
NERVOUS SYSTEM; A Manual of Diseases of the. By W. R. Gowers. 12/6 *Churchill.*

### Library of Biography and History.

BACON. By R. W. Church (Dean of St. Paul's). 5/- *Macmillan.*
GLADSTONE; Anecdotes of. By an Oxford Man. 1/ *Hamilton.*
GREECE: A History of. Part I. By Edwin Abbott. 10/6. *Rivingtons.*
HENRY II. By Mrs. J. R. Green. 2/6. *Macmillan.*
MITCHELL: Life of John. By Wm. Dillon. 2 vols. 21/- *Kegan Paul.*

### Library of Christian Literature.

A CENTURY OF CHRISTIAN PROGRESS AND ITS LESSONS. By J. Johnston. 3/- *Nisbet*
FAITHFUL DEPARTED; The Present State of the. By Rev. Geo. Body. 1/- *Masters.*
ST. JOHN, THE AUTHOR OF THE FOURTH GOSPEL. By H. H. Evans. 6/- *Nisbet.*
THE VICTORY OF THE CROSS. By B. F. Westcott. 3/6 *Macmillan.*

### Dictionaries and Encyclopædias.

EVERYBODY'S POCKET CYCLOPÆDIA. By E. Sheldon. 6d. *Saxon and Co.*
MIDDLE ENGLISH: A Concise Dictionary of. By A. L. Mayhew—W. W. Skeat. 7/6 *Froude.*
NATIONAL BIOGRAPHY; Dictionary of. By Leslie Stephens 15/- *Smith and Elder.*
NEW ENGLISH DICTIONARY ON HISTORICAL PRINCIPLES. Edited by James Murray. Part 4 (Bra-Cass). 12/6 *Froude.*

### Library of Fiction.

A BITTER REPENTANCE. By Lady Virginia Sandars. 3 vols. *Hurst and Blackett.*
A WOMAN'S FACE. By F. Warden. 3 vols. *Ward and Downey.*
CHILDREN OF GIBEON. By Walter Besant. 2/- *Chatto and Windus.*
EVE. A Romance. By Author of "John Herring." 2 vols. *Chatto and Windus.*
JESS. By H. Rider Haggard. 2/6 *Smith and Elder*

### To Residents, Secretaries, and Matrons.

THE Editor will be glad to have the Regulations and each Report as issued by Asylums, Hospitals, Convalescent and Nursing Institutions, as well as those of other Charities, and an early copy in duplicate of all Appeals and Festival Notices, etc., addressed to *The Editor, The Lodge, Porchester Square, London, W.* Any superintendent, secretary, matron, or other chief official who regularly sends such information may be registered, on application to the Publisher, to receive free of cost a cover for binding THE HOSPITAL in half-yearly volumes.

### Notice to Advertisers and Subscribers.

All Advertisements and Business Communications should be addressed to The Publisher, *not to the Editor*, THE HOSPITAL, 38, Old Jewry, E.C.
A Scale of Charges will be found in the Advertisement Sheets, page iii.

### ROYAL HOSPITAL FOR DISEASES OF THE CHEST, CITY ROAD.

The Working Men's Committee, in aid of this Institution, have arranged to hold a public meeting in St. Clement's Hall, Lever Street, City Road, on Wednesday, 29th inst., at Half-past Eight p.m., when the chair will be taken by Mr. Nicholson, President of the Mile End New Town Working Men's Society.

### THE STUDENT'S COLUMN.

### ROYAL COLLEGE OF SURGEONS.

THE following gentlemen, having passed the necessary examinations for the diploma, have been admitted members of the College :—

*Abbott, Francis Charles*, L.R.C.P.L., 14, Cowley Street, Westminster.
*Acton, Charles James*, L.R.C.P.L., 15, Scarisbrick Street, Southport.
*Appleton, Enderby James*, L.R.C.P.L., 1, Victoria Road, Old Charlton.
*Baker, Arthur Ernest*, L.R C.P.L., 28, Camden Street, N.W.
*Barnett, John Edward Sewell*, L.R.C.P.L., 20, Ditchling Road, Brighton.
*Bedford, Charles Henry*, M.B., C.M., Edin., Edinbain, Skye.
*Belcher, Henry Edward*, L.R.C.P.L., 23, Grafton Place, N.W.
*Bell, Frederick*, L.R.C.P.L., 6, Winckley Square, Preston.
*Blachford, James Vincent*, L.R.C P L., 9, Laurel Grove, Penge, S E.
*Bodilly, Reginald Thomas Harker*, L.R.C P.L , 12, Belitha Villa, N.
*Bremner, Ramsay Allan*, L.R.C.P.L., 26, Penge Road, South Norwood.
*Brown, Francis Joseph*, L.R.C.P.L., 16, Mornfield Road, W.
*Brown, Walter Sigismund*, L.S.A., Guy's Hospital.
*Brownlow, George Percy*, L.R.C.P.L., Alexandra Road, Manche ter.
*Bryett, Lewis Thomas Fraser*, L.R.C.P.L., 4, Girdler's Road, W.
*Buchanan, Robert James M'Lean*, L.R.C.P.L., 23, St. Albans Road, Bootle.
*Bullock, Charles Henry*, L.R.C P.L , 59, Fortess Road, N.W.
*Burchell, Ernest*, L R.C.P.L., 2, Kingsland Road, E.
*Cameron, Robert Watson*, L.R C.P.L., 140, Cecil Street, Manchester.
*Capron, Henri John*, L.R.C.P L., 4, Tyndall's Park, Clifton.
*Carpenter, Percy Tranto*, L.R.C.P.L , 142, Portsdown Road, W.
*Carter, Weldon, Cragg*, L.R.C.P.L., Old Dalby Grange, near Melton Mowbray.
*Caudwell, Francis Bernard Henry*, L.R C.P.L., St. Matthias Vicarage, Stoke Newington.
*Colborne, George*, L R.C.P.L., 58, Loudoun Road, N.W.
*Dallewy, John*, L.R.C.P.L., Frodsham, Cheshire.
*Davey, Samuel*, L.R C.P.L., 373, Coldharbour Lane.
*Davidson, Frederick William*, L.R.C.P L., Westminster Hospital.
*Davis, Cyril Stephen*, L.R.C.P.L , St. Aldwyns, Lewisham Park.
*Davis, Henry*, L.R.C.P.L., 136, Stanhope Street, N.W.
*Duer, Charles*, L.R.C.P.L., 22, Harewood Square, N.W.
*Duncan, Percy James*, L.R.C.P.L., 51, Great Marlborough Street, W.
*Dyer, John Edward*, L R.C.P.L., University College Hospital.
*Edmonstone, Arthur*, L.R C.P.L., 8, King's Road, Norbiton.
*Elias, James*, L.S A , Senny Bridge, Brecon.
*Ensor, Charles William*, L.R C.P.L., Wimborne, Dorset.
*Evans, George Edward Alfred*, L R.C P.L., Netherhayes, Seaton, Devon.
*Exley, John* L.K.Q C.P.I., 13, Sardinia Street, Hunslet, Leeds.
*Falkner, Edgar Ashley*, L.R C.P.L., Middlesex Hospital.
*Field, Edgar Alfred*, L.R C.P L , 5, Edge Hill, Wimbledon.
*Fox, James Stanilaus*, M.B.C.M.Ed., Crowley Hill, St.Helen's, Lancashire.
*Freer, Gerald Dudley*, L R.C.P.L., The Lawn, Stourbridge.
*Frost, John Kingdon*, L.S.A., Dunheved House, Saltash.
*Fryer, George Ernest*, L.S.A., Bowdon, Altrincham.
*Furber, Edward Price*, L.R.C P.L., 6, Upper Hamilton Terrace, W.
*Giles, Arthur Edward*, M.B. Vic Univ., 86, Tyrwhitt Road, S.E.
*Goldney, Arthur George Nelson*, L.R.C.P.L., 13, Brook Gr., Hammer.mith.
*Goodfellow, Thomas Ashton*, L.R.C.P.L., Hatherton, near Stockport.
*Gordon, William*, L.R.C.P.L., 25, Bernard Street, Russell Square.
*Greville, Sampson John Rodger*, L.R.C.P.E., 4, Finnart Tr., Greenock, N.B.
*Grey, John Temperley*, L.R.C.P.L., Cotham Park, Bristol.
*Hancock, John Edwin*, L.R C.P.L., Callington, Cornwall.
*Handcock, George*, L.S.A., Hunslet, Leeds.
*Harris, George Victor Sampson*, L.R.C P.L., 30, Strathblaine Road, Clapham Junction.
*Harvey, Frank*, L.S.A., 1, Oakleigh Terrace, Plymouth.
*Haviland, Frank Papillon*, L.R.C.P.L., 37, Warrior Sq., St. Leonards-on-Sea
*Higgins, Hubert*, L.R.C.P.L., La Huerta, Wallington.
*Hill, Robert*, L.R.C.P.L., 22, Mecklenburgh Square, W.C.
*Hosking, John Edward Francis*, L.R.C.P.L., 25, Montem Road, S.E.
*Housman, Basil Williams*, L.R.C.P.L., Perry Hall, Bromsgrove.
*Hughes, Samuel Henry*, L.R.C.P.L., Woollora, Shortlands, Kent.
*Huxley, Henry*, L.R C.P.L., 4, Marlborough Place, N.W.
*Jowers, Lancelot Emilius*, L R.C.P.L., 1, Barnard's Inn.
*Kearney, James*, L.S.A., 108, Stockwell Road, Brixton.
*Keller, Otto Eugene*, M.B., Berne, German Hospital, Dalston.
*Kingston, Percy John*, L.R.C.P.L., 31, Sutherland Avenue, W.
*Laing, Alfred William*, L.R.C.P.L., West Hill, Putney.
*Langdale, Henry*, L.R.C.P.L., Rusholme, Manchester.
*Langridge, Frank Washington*, L.R.C.P.L., 35, Dulwich Road, S.E.
*Larcombe, George Garmany*, M.D , Bellevue, 25, Hogarth Road, S.W.
*Larkham, Edward Thomas*, L.R.C.P.L , 69, St. John's Road, Highgate.
*Leman, Thomas Curtis*, L.R.C.P.L., Chipping Sodbury, Gloucestershire.
*Lewis, Charles Harvey*, L.R.C.P.L., Meopham Vicarage, Gravesend.
*Lockett, John Arthur Pope*, L R.C.P.L., 165, Victoria Park Road, E.
*Lomas, Ernest Courtney*, M.B., Vic. Univ., Withington, Manchester.
*Maclure, Herbert William*, L.R.C.P L., 17, Westbourne Park Terrace.
*M'Cardel, Edward John*, M D., Kingston, 51, Torrington Square.
*M'Cullagh, Richard Chiveley*, M D.R.U I., West Cliff Grdns., Bournemouth
*Martin, Charles Lister*, L.R.C.P.L., 22, Kidbrooke Gardens, Blackheath.
*Midelton, William John*, L.R.C.P.L., 31, Sutherland Avenue, W.
*Moss, Arthur James*, L.S.A., 11, Royal Crescent, Whitby.
*Murray, George Redmayne*, L.R C.P.L., 65, Green Street, W.
*Newton, Sidney Frederick*, L.S.A., Crofton, Fareham, Hants.
*Oakman, Joseph John*, L.S.A., The Priory, Battersea.
*O'Brien, Philip Kennedy*, L.R.C.P.L., 71, Clyde Road, Croydon.
*Ogle, John Gilbert*, L.R.C.P.L., 7, Camden Street, N.W.
*Oldham, Benjamin Curwen*, 70, Avonmore Road, W.
*Ozzard, Fairlie Russell*, L.R.C.P.L., 1, Hampton Road, Forest Gate.
*Parkin, Alfred*, L.R.C.P.L., High Town, Liversedge.
*Part, John Shepley*, L.R.C.P.L , 25, Ashchurch Park Villas, W.
*Pearson, James*, L.R.C.P.L., 37, Bank Road, Liverpool.
*Pennell, George Herbert*, L.R.C.P.L., Guy's Hospital.
*Pierce, Bedford*, L.R.C.P.L , 8, Union Road, N.
*Platt, James Robert*, L.R.C.P.L., 16, De Montfort Street, Leicester.
*Platt, John Edward*, L.R.C.P.L., Upper Mill, near Oldham.
*Pogson, John William Buckley*, 95, Albert Road, Aston.
*Poulter, Arthur Reginald*, L.R.C.P.L., Shortlands, Kent.
*Powell, William*, L.R.C.P.L., 136, Acre Lane, Brixton.
*Pringle, James Hogarth*, M.B.Ed., Torquhan Stow, Midlothian, N.B.
*Pym, Charles Brownlow*, L.R.C.P.L., Manor Road, Wallington.
*Richards George Oliver*, 33, Paradise Street, Birmingham
*Roberts, John William*, L.R.C.P.L., Dolenoz, Llanidloes, Montgomeryshire
*Robinson, Edward Stanley*, L.R C.P.L., Stourport.
*Rudd, William Arthur*, L.R.C.P.L., 186, Amherst Road, E.
*Sehnebage, Caleb*, M.B.Ed., 120, Fenton Street, Leeds.
*Sclater, Nelson Came on*, L.K.Q.C.P.I., 3, Westmoreland Road, Liscard.
*Shells, William Francis Michell*, L.R.C.P.L., 4, Farm Avenue, Streatham.
*Sims, George Samuel*, L.R.C P L., 644, Commercial Road, E.
*Skinner, George Henry*, L.R.C.P.L., 29, Collingwood Road, Bristol.
*Skyrme, Henry Edward*, L.R.C.P.L., St. Arvan's, Chepstow.
*Smith, Harry Lyon*, Chester-le-Street, Durham.
*Speedy, Robert George Dunell*, L.R.C.P.L , 51, Queen Anne Street, W.
*Stephens, James William*, L R.C.P.L., Kilgerran, R.S O. South Wales.
*Summerskill, William* L.R.C.P.L , 6, Kingston Place, Leeds.
*Sunderland, Oliver*, L.R.C.P.Ed., Bexley Heath, Kent.
*Sylvester, Harold Augustus*, L.R.C.P.L., 1, Barnard's Inn, E.C.
*Tate, Walter William Hunt*, L.R.C.P.L., 2, The Terrace, N.W.
*Tench, Montague*, L.R C.P.L., 2, Edith Road, W.
*Thomas, Archibald*, L.S.A., 42, Portland Road, W.
*Tressider, William Elliot*, L.R.C.P.L., Alleyne Park, West Dulwich.
*Turney, Horace George*, L R.C.P.L., 198, Camberwell Grove, S.E.
*Vallancy, Aymer d'Estampes de*, L.R.C P.L., 5, Tavistock Crescent, W.
*Verdon, Francis*, L.R.C.P.L., 37, Craven Street, Charing Cross.
*Wade, Charles*, L.R.C.P.L., Boscastle, Cornwall.
*White, Edgar Ramsay*, L R.C.P.L., 4, Hazelside Road, N.
*White, Joseph Farrell*, L.R.C.P.L., Fairfield, Manchester.
*Wilkins John*, L R C.P.L., 43, Wolseley Road, N.
*Wilkinson, Edmund*, L.R.C.P.L., 15, Queen's Terrace, Hammersmith.
*Williams, Charles Louis*, M.B.C.M.Ed., 8, Princes Terrace, Birkenhead.
*Willoughby William George*, L.R.C.P.L., 14, Downshire Hill, Hampstead.
*Wills, Ernest*, L.R.C.P.L , 33 Union Street, Torquay.
*Wilson, Charles*, L.R.C.P.L., Cambridge Road, E.
*Woodhams Sidney*, L.R.C.P.L., Tordon Road, Ealing.
*Wynne, John Darley*, M.B., Dublin Public Hospital, Manchester.

One hundred and twenty-five candidates were referred to their professional studies.

---

### GOOD-NATURED COMMITTEES.

IN one large home on our south coast, which specially denounces and forbids those who require medical or surgical treatment, the committee lately granted £70 to the resident physician for a case of surgical instruments. They really were beautiful instruments, and the committee enjoyed looking at them when they paid their annual visit. Some of the subscribers to the home may perhaps have thought the expenditure unwarrantable, as there is not the least likelihood of any of these instruments ever being required. But then of course an accident might happen, and it is always well to be prepared for the worst. This is true; but then the same committee have appointed a lady as matron who is not a trained nurse: if ever those instruments are used, who is to tend the poor victim who suffers? The whole fault here lies in too good-natured a committee who have put their hand to a good work, as they well know, but who do not concern themselves enough about the carrying on of the same. That is why we would ask those who now happen to be near a home to pay a casual but thorough visit of inspection, and try and see things in their everyday aspect. Because a work is good is no reason why it should not be better, and the fault which keeps hundreds of people from profiting by the benefits of convalescent homes is one which might surely be remedied. For once a patient does get through the guarded portals, a pleasant enough life awaits him, and joyous indeed is the freedom from care, and the long days spent by the health-giving sea. Surely paterfamilias, as he lies on the beach watching his sturdy youngsters paddling in the water or racing on the sands, will give a thought to the wistful pale faces peering forth from the London smoke, waiting patiently for a vacancy in some convalescent home. One of the last duties before going off for a holiday should be to send a cheque to the Samaritan Society. But even during the holidays we have our duties, and the man who will not only give his money, but also his consideration to this subject of convalescents, will be in truth a neighbour to his fellow men. More homes and better are what we badly need—not to take the place of those already existing, but to fill up the gaps between them. Let those who are enjoying their holidays not forget that many thousands of persons who are sick and weak have yet to go toiling on because there are not enough holiday homes.

### Amusements with Profit for all Ages.

**Rules.**—All answers must be addressed to the Prize Editor, The Hospital, 38, Old Jewry, Cheapside, London, E.C., not later than the *first* post on Thursday next. The full name and address must in all cases be given, but a *nom de plume* may be added for publication. Only one side of the paper must be written on.

### FOR MEN AND WOMEN.

**Rule.**—Competitors can enter for *all* quarterly competitions, but no competitor may take more than *two quarterly prizes* during the year.

### First Quarterly Poetical Competition.

Commenced May 26th, 1888; ends August 25th, 1888.

This quarter a PRIZE of ONE GUINEA will be given to the competitor who gains the highest number of marks for original poems, on any subjects suitable for insertion in this paper. All contributions sent in will be credited according to merit, eighteen marks being the highest score for any one contribution. This competition to alternate weekly with the set acrostic competition.

### Acrostic Competition.

This quarter the original acrostic competition will be discontinued, and A PRIZE of HALF-A-GUINEA will be given to the competitor who gains the highest score for solution of acrostics set. *One mark* only will be given to the competitors who solve all the lights of each acrostic.

Exception will be made in the case of *quotation acrostics*, when *two marks* will be given, *one* for the correct solution of all *lights*, and *one* mark for the *source* of the quotations.

This week credit will be given for

### No 7.—Original Poem.

### Rest.

When the sun sinks in the skies,
Then the labourer's toil is o'er,
Gladly to his home he hies,
Time to rest has come once more.

What a blessed time is night!
Hushing every busy sound,
Shrouding nature from our sight,
Giving rest to all around.

Hours of toil may fill the day,
But the even comes apace,
Rest will help us on our way,
Cheer us in our earthly race.

On creation falls a hush;
All are wrapped in sweet repose,
Silence reigns in every bush,
Even little flowerets close.

Now has come the time for sleep—
Rest for man, and bird, and beast,
Till the sun begins to peep
In the glorious golden east

But the brain, opprest by day,
Strives to rest, and strives in vain;
Then we hear the thinker say—
"Would it were the day again!"

And when day shall dawn once more,
If he have learnt his lesson well,
Body shall work hard as before,
But brain shall rest a little spell.

So, when next he seeks his bed,
Longing for a slumber light,
All his phantoms will have fled,
For he has used his day aright. —*Patience.*

### Results of No. 6 Original Poem.

Dodo (16), A. M. E. (16). Anthem (15), Patience (14), Hesperomis (13), S. M. W. (13).

### THE CHILDREN'S CORNER.

PRIZES.
FOR MOST CORRECT ANSWERS TO PUZZLES.
This Commenced October 1st, 1887.
One Pound a Month.

A PRIZE OF THREE GUINEAS

to the Competitor who shall have sent in the largest number of correct or best answers during the twelve months.

### Fourth Week Puzzles.

No. 13. Biographical Acrostic.—1. Three Roman brothers. 2. An eminent Irish politician and orator. 3. Wise men of the East. 4. Wife of Etheldred II. 5. A noted English outlaw. Initials give the name of a Greek writer, finals his chief work.

No. 14. Missing Word Puzzle.—Fill in the blanks and give the name of the Author:—

Close — — hounds — — came
— cheer — — the — —
But, ——— — the — —,
— gallant —— fell.
The —— — strove — —
— rouse — — — — and —,
For — — steed, —— —— ——
— his — —, to — — —;
Then — — pity — —
— —— o'er — — —.

No. 15. Arithmetical Puzzle.—A man buys a pair of boots valued at 25*s.*, in payment for which he tenders a £5 note. The bootmaker goes to his neighbour, a baker, to change the note. The customer, having received £3 15*s.* change, departs with the boots. Presently the baker comes to the bootmaker to say that the note is worthless; and the bootmaker returns him £5. What does the bootmaker lose?

No. 16. Charade.—My *First* is vain, my *Second* strong, my *Whole* a weed.

### Results of Second Week Puzzles.

No. 5. Historical Mental Picture.—The Prince was Robert Duke of Normandy, his brothers were William II. and Henry I., and his father was William I.

No. 6. Arithmetical Puzzle.—The father was 37, the sons 17 and 3; the eldest son was born in 1870.

$$1887 \div 3 = 629 \div 17 = 37.$$

$$\begin{array}{r} 1887 \\ 17 \\ \hline 1870 \\ \hline \end{array}$$

No. 7. Double Acrostic

K enilwort H
E br O
N a P
T hebe S

No. 8. Enigma. A pair of compasses.

| Marks. | August 11th. | Total 1st and 2nd Weeks. |
|---|---|---|
| A. C. K. | 4 | 8 |
| Alsie | — | 1 |
| Boadicea | 3 | 7 |
| Curly | — | 4 |
| Hercules | 2 | — |
| Poodle | — | 2 |
| Thanet | 4 | 8 |
| Scrooge | 4 | 8 |
| Gem | 3 | 7 |
| Ally | 3 | 7 |
| R. H. | 3 | 7 |
| G. M. M. | 3 | 7 |
| Janette | 4 | 7 |
| Tynesider | 3 | 6 |

### Conditions.

I. Every paper sent in must be clearly written, and one side only must be used. All competitors must mark at the head of their papers the number of their competition.

II. Each paper must be signed by the author with his or her real name and address. A *nom de plume* may be added, if the writer does not desire to be referred to by us by his real name. In the case of all prizewinners, however, the real name and address will be published.

III. All communications relating to prize competitions should be addressed to "The Prize Editor, The Hospital, 38, Old Jewry, London, E.C." Competitors are requested not to include in letters, etc., to the Prize Editor communications on matters of other business. All letters must be fully prepaid.

IV. The Prize Editor of The Hospital is the sole judge of the competitions, and his decision is in all cases final and without appeal.

V. The Prize Editor reserves the right to publish any paper sent in, whether it receives a prize or not. He does not hold himself responsible for any MSS. sent, nor can he undertake to return rejected competitions.

VI.—All children resident at school are requested to forward their *home* address in addition to the school one, when entering for the "Children's Corner" Competitions.

**Notice to all Correspondents.**—N.B.—All letters referring to this page, which do not arrive at 38, Old Jewry, Cheapside, London, E.C., by *the first post on Thursdays*, and are not addressed PRIZE EDITOR, will in future be disqualified and disregarded.

No one will be permitted to win the Monthly Prizes twice in any one year, the Annual Prize being offered especially to prevent this.

